# Pre-Outplacement Perceptions of Dental Students Regarding Rural Oral Health Practice and Associated Factors

**DOI:** 10.3390/dj8010022

**Published:** 2020-02-23

**Authors:** Menaka Abuzar, Felicity Crombie, Karin Bishara, Annesley Bryan, Kenneth Chan, Brendan Chang, Denise Chang, Wei-I (Elizabeth) Cheng, Ryan Chu

**Affiliations:** 1 Melbourne Dental School, University of Melbourne, Melbourne 3010, Australia; fcrombie@unimelb.edu.au (F.C.); kbishara@student.unimelb.edu.au (K.B.); abryan@student.unimelb.edu.au (A.B.); kennethc2@student.unimelb.edu.au (K.C.); brendanc@student.unimelb.edu.au (B.C.); xchang@student.unimelb.edu.au (D.C.); rchu@student.unimelb.edu.au (R.C.); 2 School of Dentistry and Oral Health, Griffith University, Gold Coast 4215, Australia

**Keywords:** first-year dental students, rural oral health, rural dental training, rural affiliation

## Abstract

Rural outplacement programs have been offered by Australian universities to encourage dental students to consider rural careers. The dental curricula should be designed to provide graduates with a good understanding of oral health issues that rural oral health care workers face. Pre-outplacement perceptions of dental students on rural practice are considered imperative to review and update the outplacement programs; however, they have not been investigated in detail. An online, anonymous, voluntary survey was conducted among the first-year dental students. The responses were solicited on the level of awareness and understanding of rural oral health, intention to practice in rural locations and factors informing the appeal of rural dental practice. The mean of a seven-point Likert scale revealed that most students had some level of perceived knowledge and awareness of rural communities and oral health. Students with rural affiliation were more likely to have the intent to practise rurally after graduation (*p* = 0.001). For short-term rural practice, students overall expressed positive intention. Greater job opportunities were the main motivating factor, while the distance from family and friends was the main deterring factor for practising rurally. Rural affiliation influences the intent to practise rurally on graduation. The pre-outplacement perceptions, in this study, are comparable with post-outplacement observations reported in the literature.

## 1. Introduction

Dental outplacements have become an integral part of clinical training in many dental schools around the world [1,2,3]. Outplacement programs have been introduced with the view of explicit beneficial outcomes for students and institutions as well as communities [1,4,5]. Students benefit through a broad range of clinical experiences and the opportunity to work as a team. Educational institutions benefit as the clinical facilities and teaching staff are resourced and managed by host organizations. Communities benefit from the services that dental students provide. Dental outplacements in ‘rural’ areas have been established with an added strategic objective to attract future dentists to rural areas [6,7,8,9].

The stark disparity in the distribution of dentists between rural and urban locations has been reported in many countries. In Australia, about 63 dentists per 100,000 persons practise in major cities, whereas, this number is about 26 in Remote/Very Remote areas [10]. The shortage of dentists has an adverse effect on the oral health of rural communities and their quality of life [11]. There are significant barriers to oral health care professionals working in rural and remote areas, for example, social isolation, insufficient remuneration, accessing appropriate educational facilities for children and lack of opportunities for professional development [12,13]. Several strategies have been adopted to address the shortage of practicing dentists in rural areas including programs of rural outplacement training for dental graduates. These programs are designed to provide rural experiences to students, on the premise that health care graduates and professionals, with a rural background or awareness, are more likely to work in rural areas [14,15,16,17].

In the last two decades, several dental schools around the world including Australia have introduced rural outplacements to provide students with clinical experience in a rural community and to acquaint students with the rural environment [16]. The post-placement perceptions of these students have been generally positive regarding their rural clinical experience, their increased understanding of rural oral health and their intentions to pursue rural practice after graduation [7,8,9]. The Australian Dental Council [18] requires graduates to be competent in servicing rural and Indigenous populations. Consequently, it is mandatory that the dental curriculum is designed to provide relevant knowledge of rural clinical practice and an overall rural experience to students. To meet this goal, it is important to continue to review and update the curricula to ensure students achieve their learning outcomes. One important facet of this review is to assess students’ knowledge and understanding of rural communities at an early stage in the program and prior to the rural out-placement. Anecdotal evidence indicates that positive feedback from senior students may have an impact on the cognizance of students in the early years of the program concerning rural outplacements. However, little has been studied so far regarding the pre-outplacement perceptions of dental students [16]. This paper presents the results of a pre-outplacement surveys of first-year dental students from an Australian university, which continues to convene a well-established rural outplacement program since 2006 [7,19].

This study aimed to assess the following in a cohort of first-year dental students’: (a) Level of awareness and understanding of rural communities and rural health, (b) intention to practise in rural areas; and (c) the influencing factors for considering rural dental practice. The association between students’ perceptions and ‘rural affiliation’ was also investigated. The results of this survey have been discussed with reference to post-placement results, published in the literature.

## 2. Materials and Methods

The first-year Doctor of Dental Surgery (DDS) students at the University of Melbourne participated in an anonymous, voluntary, online questionnaire delivered via SurveyMonkey^®^ (Survey Monkey Inc., San Mateo, CA, USA) during a compulsory class in March 2018. Ethics was approved by the Human Ethics Advisory Group (HEAG) Committee, Melbourne Dental School, University of Melbourne (Ethics ID: 1647795.1, 17 July 2017).

The survey consisted of multiple questions on demographic attributes, perception of rural environment, understanding of rural oral health and practice, the intention of rural practice and factors affecting their work intention. The responses were solicited on a seven-point Likert scale, where 1 denoted ‘entirely disagree’ and 7 denoted ‘entirely agree’ [20]. One qualitative question investigated barriers to long term rural practice. The responses to this open-ended question were thematically coded by seven investigators and the data were compiled and analysed using an Excel spreadsheet (Microsoft^®^). Perceptions of rural understanding were solicited before providing participants with a map of the Australian Standard Geographical Classification [21] of remoteness as a guide. The term ‘rural’ was used synonymously with ‘regional’. Participants were also asked to rank their preferred location of future practice - major city, rural/regional or remote.

Survey data was analysed using SPSS^™^ Version 24 (IBM^®^, Armonk, NY, USA) software. The missing responses for individual questions were excluded from the analysis. In addition to the direct responses for individual questions, two composite measures were also generated for the analysis—‘rural affiliation’ and ‘rural knowledge’. ‘Rural affiliation’ was established on the current or past rural residential status, place of birth and the association with family or friends in a rural area. ‘Rural knowledge’ was defined using the average response of six questions on rural understanding and perception. Responses to individual questions were analysed using descriptive statistics. Mann-Whitney U Test was used to explore whether ‘Intentions to work in rural areas’ (ordinal response) differed significantly based on gender, age group, ‘rural knowledge’ or ‘rural affiliation’ (binary attributes).

## 3. Results

Of the total 101 first-year dental students, 94 submitted their responses (85.5% response). Most participants (80.9%) were under the age of 24. Female representation was slightly higher (51.1%).

Students reported having minimal knowledge and awareness of the rural environment as shown by the average score of their responses on the 7-point ordinal scale (Table 1). They expressed to know the meaning of ‘rurality’ (Mean ± SD: 5.38 ± 1.29), but were unsure of the difference between rural, regional and remote (4.29 ± 1.56). They expressed low confidence in their ability to interact socially and professionally with rural and Indigenous communities (Mean ≥ 4.47 ± 1.51) and no confidence of Indigenous culture knowledge (3.62 ± 1.49). On average students were somewhat aware of the oral health issues and dental practice challenges in rural areas (Table 2). They agreed with the lack of, and need for, dental specialists in rural compared to metropolitan areas (5.78 ± 1.07; 5.65 ± 1.23, respectively).

Students identified specific professional and personal factors that influence the decision on rural practice (Table 3). They generally perceived ‘strong living support’, ‘strong financial incentives’ and ‘proximity of friends and relatives’ (Mean ≥ 5.23) as the key positive factors in rural practice option. Further, while responding to the specified question with multiple choice, they considered factors such as ‘greater job opportunities’ and ‘greater remuneration’ as motivators to rural practice (Table 4). The main barriers perceived, as per open-ended responses, were personal, such as distance from family/friends and lifestyle differences (Table 5).

Regarding rural clinical training (Table 6), students on average agreed with the importance of involvement in rural outplacement programs and curriculum focus on rural issues and rural oral health (Mean ≥ 5.27).

When asked about their preferences for the location of their practice after graduation, most students gave their first preference (92%) for a city, the second preference (82%) for rural and the third (85%) for remote areas.

On the question of their interest in working in a rural area after graduation (Table 4), the overall response was neutral (3.99 ± 1.61). For the long-term rural practice, the average response was negative (3.70 ± 1.51) but they were mostly positive about short-term rural practice (5.46 ± 1.3).

Table 7 presents the association of dental students’ work intention in a rural area with their demographic attributes. There was no significant difference in their intention for rural practice due to their gender, age group or their current rural knowledge (*p* > 0.2). However, there was a significant difference due to ‘rural affiliation’ for their interest in rural practice after graduation (*p* = 0.001), as well as their consideration for long-term practice (*p* = 0.002). Overall, students did not differ significantly for their positive inclination towards short-term rural practice irrespective of any attributes (*p* > 0.15).

## 4. Discussion

One of the key recommendations of the National Oral Health Plan, Australia, is to enhance programs to recruit and retain dental practitioner students in regional and rural areas [22]. To this effect, it is suggested that the proportion of hours spent in rural placements, and the number of students enrolled in dentistry programs from rural and regional areas, has to be considered. Currently, the number of rural students enrolled in dental programs remains low [23]. In this study too, there was a small number of respondents with a ‘direct’ rural or Indigenous background. However, when we considered the less ‘direct’ rural background, about one-third of our sample was found to have some level of ‘rural affiliation’.

In the absence of comparable studies, the results of this study are discussed in the context of published literature relating to post-outplacements. The items compared and discussed are the influence of rural background and its association with intention to work rurally (long and short-term), perceived barriers for considering working rurally and knowledge of Indigenous culture.

Similar to an earlier study with first-year medical students [24], dental students with ‘rural affiliation’ in this study were more likely to consider long-term practice in rural areas. A systematic review of rural work movements [25] identified previous experience in rural areas as the most influential motivational factor for rural recruitment and retention, which is similar to the findings of this study. Social and lifestyle factors are also identified as important for both short and long-term retention of medical and allied rural health practitioners [26,27]. The results of our study also demonstrate proximity to family and friends was perceived to be an important factor for considering long-term rural practice, which is comparable with post-outplacement surveys [6,7,8]. Hence, incentives for financial and professional opportunities alone may not be adequate to address the issue of long-term retention of rural dental practitioners. Short-term rural practice was considered by most students irrespective of their ‘rural affiliation’. Greater job opportunities and greater remuneration were the most cited influencing factors by this cohort of first-year dental students in considering short-term rural practice.

The mandatory inclusion of appropriate rural and Indigenous learning content in undergraduate curricula in Australian dental schools has taken place through government support for rural placements which are adequately resourced. However, it is equally important to continue to support new graduates through mentoring programs organised through educational institutions and professional organisations (e.g., Australian Dental Association) when they choose to practice in relatively smaller rural and Indigenous communities.

Participants in this pre-outplacement survey expressed low confidence in their knowledge of Indigenous culture. In an earlier study, medical students’ post-rural-outplacement surveys indicated they hold stereotypical views of Indigenous people and Indigenous health status, and they were not completely prepared for Indigenous health work [28]. The results from a dental post-outplacement survey indicate the outplacement experience improved students’ understanding of Indigenous issues [19]. This may be attributed to the fact that the rural outplacement included clinical experience at an Indigenous clinic where students had the opportunity to interact with Indigenous staff. Interpretations can be made with caution that the rural outplacements are valuable in improving students’ knowledge of Indigenous culture. Definite conclusions will be possible in the future by comparison of post-placement information from this same cohort of students.

The first-year dental students who responded to this survey indicated low confidence in knowledge of the rural environment. Interestingly, they were positive about their perceived knowledge of rural oral health and of rural dental practice (‘somewhat agree’, mean of 5 in a 7-point Likert scale). The participants of the survey had no or minimal formal teaching in rural and Indigenous content in the dental program at the time of the survey. The positively perceived knowledge may be attributed to the following factors: Increased public awareness of rural affairs in the last decade, less employment opportunities in metropolitan areas for new graduates and students’ self-preparedness to work rurally on graduation, and post-placement feedback from senior students regarding their mandatory rural outplacement program experience.

Studies like this, which are based on survey campaigns, may be affected by participation bias. Social environment and the context of a particular survey may lead students to give what they perceive as socially-desirable responses. Therefore, caution should be taken in interpreting the results.

Since this study is based on one cohort of students from one dental school, the results may not reflect the general perceptions of other cohorts across other schools. Therefore, further investigations involving other dental schools in Australia are warranted. Follow-up studies of future cohorts, with pre-outplacement in the early years of the dental program and post-outplacement experience in the final year, would provide evidence to better evaluate the outcomes concerning students’ perceptions of rural understanding, intention for rural practice and the effectiveness of the curriculum.

## 5. Conclusions

The pre-placement perceptions of dental students have notable similarities with those of post-placement programs in the literature. First-year dental students expressed some awareness of the issues affecting rural communities concerning oral health. They reported low confidence in the knowledge of Indigenous culture. Students with rural affiliation were more interested in practising rurally long term than those without an affiliation. However, students overall did not differ significantly in expressing their positive inclination towards short-term rural practice irrespective of any attributes (*p* > 0.15) i.e., gender, age-group, rural knowledge or rural affiliation. Influencing factors for consideration of rural dental practice by first-year dental students are related to financial and professional incentives. The factors deterring students to stay long-term in rural communities are centred on proximity to family and friends.

## Figures and Tables

**Table 1:** First-year dental students’ perception and understanding of rural environment.

Questions	Mean ^†^	SD ^†^
▪ I have a clear understanding of what rurality means.	5.38	1.29
▪ I understand the difference between rural, regional, and remote.	4.29	1.56
▪ I am confident in my ability to socially interact with rural communities.	4.82	1.54
▪ I am confident in my ability to professionally interact with rural communities.	4.96	1.42
▪ I am confident in my ability to socially interact with Indigenous Australians.	4.47	1.51
▪ I am confident in my ability to professionally interact with Indigenous Australians.	4.71	1.44
▪ I am confident in my knowledge of Indigenous culture.	3.62	1.49

N = 94. ^†^ Mean and standard deviation (SD) of the 7-point Likert scale values, which are: 1 = ‘Entirely disagree’, 2 = ‘Mostly disagree’, 3 = ‘Somewhat disagree’, 4 = ‘Neither agree nor disagree’, 5 = ‘Somewhat agree’, 6 = ‘Mostly agree’, and 7 = ‘Entirely agree’.

**Table 2:** First-year dental students’ perception of dental practice and oral health in rural areas.

Questions	Mean ^†^	SD ^†^
▪ Working as a rural dentist is more complex than working as a metropolitan dentist.	5.01	1.14
▪ Communication is more challenging in rural dentistry than in metropolitan dentistry.	4.70	1.21
▪ Rural dentists have less professional support than metropolitan dentists.	5.33	1.07
▪ A general dentist working rurally has to have broader dental knowledge than a general dentist working in a metropolitan area.	4.61	1.34
▪ There are fewer dental specialists working rurally than in metropolitan areas.	5.78	1.07
▪ There is a need for dental specialists in rural areas.	5.65	1.23
▪ Rural residents have poorer dental health status than metropolitan residents.	5.43	1.20
▪ There is a disparity in access to dental care for metropolitan residents compared to rural residents.	5.31	1.34

N = 89. ^†^ Mean and standard deviation (SD) of the 7-point Likert scale values, which are: 1 = ‘Entirely disagree’, 2 = ‘Mostly disagree’, 3 = ‘Somewhat disagree’, 4 = ‘Neither agree nor disagree’, 5 = ‘Somewhat agree’, 6 = ‘Mostly agree’, and 7 = ‘Entirely agree’.

**Table 3:** First-year dental students’ perception of the influencing factors affecting rural practice decision.

Questions	Mean ^†^	SD ^†^
▪ I would consider working rurally if I had strong living support/stable accommodation.	5.23	1.23
▪ I would consider working rurally if I had strong financial incentives.	5.54	1.07
▪ I would consider working rurally if my family and friends lived nearby.	5.56	1.17
▪ There are lifestyle advantages in working rurally.	4.31	1.50
▪ There are professional advantages in working rurally.	4.90	1.38

N = 87. ^†^ Mean and standard deviation (SD) of the 7-point Likert scale values, which are: 1 = ‘Entirely disagree’, 2 = ‘Mostly disagree’, 3 = ‘Somewhat disagree’, 4 = ‘Neither agree nor disagree’, 5 = ‘Somewhat agree’, 6 = ‘Mostly agree’, and 7 = ‘Entirely agree’.

**Table 4:** Responses to specified motivating factors for decision to practise in rural area.

Motivating factors	Count ^†^	(%)
▪ Greater job opportunities	68	28.5
▪ Greater remuneration	48	20.1
▪ Attractive rural lifestyle	35	14.6
▪ Moral responsibility	33	13.8
▪ Diverse culture	31	13.0
▪ Greater exposure to Indigenous health	24	10.0

^†^ Multiple responses.

**Table 5:** Summary of open-ended question on discouraging factors to practise in rural area.

Discouraging factors.	Count ^†^	(%)
▪ Distance from family and friends	50	49.1
▪ Distance from city	14	13.7
▪ Lifestyle differences	14	13.7
▪ Lack of access to facilities	10	9.8
▪ Lack of career development and job opportunities	7	6.9
▪ Finance related issues	4	3.9
▪ Lack of support	3	2.9

^†^ Compiled from open-ended question.

**Table 6:** Opinion of first-year dental students about the relevance of rural clinical training and their intention to work in rural areas after graduation.

Questions	N	Mean ^†^	SD ^†^
**RURAL CLINICAL TRAINING**			
▪ It is important for students to be involved in rural placement programs during their course.	94	5.68	1.31
▪ I am willing to be involved in a rural placement program during my course.	94	5.40	1.62
▪ It is important to learn about rural dental health during the DDS course.	89	5.90	0.97
▪ There should be a significant focus on rural issues within the DDS course.	89	5.27	1.10
**INTENTION FOR RURAL PRACTICE**			
▪ I am interested in practising rurally after graduating my course.	87	3.99	1.61
▪ I would consider working in a rural area for a short term (up to 2 years).	87	5.46	1.30
▪ I would consider working in a rural area for a long term (>2 years).	87	3.70	1.51

^†^ Mean and standard deviation (SD) of the 7-point Likert scale values, which are: 1 = ‘Entirely disagree’, 2 = ‘Mostly disagree’, 3 = ‘Somewhat disagree’, 4 = ‘Neither agree nor disagree’, 5 = ‘Somewhat agree’, 6 = ‘Mostly agree’, and 7 = ‘Entirely agree’.

**Table 7:** Work intention of first-year dental students for rural practice and its association with their gender, age group, ‘rural affiliation’ and ‘rural knowledge’.

			Interested in Practising Rurally After Graduation		Considering Short-Term Work in Rural (≤2 years).		Considering Long-Term Work in Rural (>2 years).	
Attributes	Binary Classes	N	Mean Score ^†^	*p* ^‡^	Mean Score ^†^	*p* ^‡^	Mean Score ^†^	*p* ^‡^
Gender	(1) Male	42	3.95	0.720	5.36	0.217	3.74	0.965
	(2) Female	44	4.09		5.64		3.73	
Age Group	(1) ≤24 years	70	3.90	0.372	5.44	0.907	3.63	0.337
	(2) >24 years	17	4.35		5.53		4.00	
Rural Knowledge ^§^	(1) With knowledge	65	3.91	0.478	5.40	0.322	3.68	0.704
	(2) No knowledge	22	4.23		5.64		3.77	
Rural Affiliation¶	(1) With affiliation	30	4.70	0.001	5.70	0.159	4.40	0.002
	(2) Without affiliation	57	3.61		5.33		3.33	

^†^ Mean of the 7-point Likert scale values: 1 = ‘Entirely disagree’, 2 = ‘Mostly disagree’, 3 = ‘Somewhat disagree’, 4 = ‘Neither agree nor disagree’, 5 = ‘Somewhat agree’, 6 = ‘Mostly agree’, and 7 = ‘Entirely agree’. ^‡^ Mann-Whitney U Test. ^§^ Average value of 7-point ordinal responses to six questions on rural understanding and perception: (1) ‘With Knowledge’ > 4; (2) ‘No Knowledge’ ≤ 4. ¶ Based on the current/past rural residential status, rural place of birth and the association with family or friends in a rural area.
